# Effect Evaluation of Spatial Characteristics on Map Matching-Based Indoor Positioning

**DOI:** 10.3390/s20226698

**Published:** 2020-11-23

**Authors:** Shuaiwei Luo, Fuqiang Gu, Fan Xu, Jianga Shang

**Affiliations:** 1 School of Geography and Information Engineering, China University of Geosciences, Wuhan 430078, China; 2834@pdsu.edu.cn (S.L.); xufan12321@cug.edu.cn (F.X.); 2 College of Tourism and Planning, Pingdingshan University, Pingdingshan 467000, China; 3 School of Computing, National University of Singapore, Singapore 117418, Singapore; fuqiang.gu@nus.edu.sg; 4 National Engineering Research Center for Geographic Information System, Wuhan 430078, China

**Keywords:** map matching, indoor positioning, spatial information, particle filtering, hidden Markov model, geometric

## Abstract

Map-matching is a popular method that uses spatial information to improve the accuracy of positioning methods. The performance of map matching methods is closely related to spatial characteristics. Although several studies have demonstrated that certain map matching algorithms are affected by some spatial structures (e.g., parallel paths), they focus on the analysis of single map matching method or few spatial structures. In this study, we explored how the most commonly-used four spatial characteristics (namely forks, open spaces, corners, and narrow corridors) affect three popular map matching methods, namely particle filtering (PF), hidden Markov model (HMM), and geometric methods. We first provide a theoretical analysis on how spatial characteristics affect the performance of map matching methods, and then evaluate these effects through experiments. We found that corners and narrow corridors are helpful in improving the positioning accuracy, while forks and open spaces often lead to a larger positioning error. We hope that our findings are helpful for future researchers in choosing proper map matching algorithms with considering the spatial characteristics.

## 1. Introduction

While the Global Navigation Satellite Systems (GNSSs), such as GPS and Beidou, have been successfully applied in plenty of tasks, it does not work well in indoor environments since the satellite signals fail to pass through obstacles (e.g., buildings). To address the limitation of GNSS, a number of indoor positioning techniques have been proposed in recent years [1,2,3,4,5,6], including Wi-Fi, vision, light, and ultra-wideband (UWB), Bluetooth, and inertial sensors. However, each technique has its own drawbacks in terms of accuracy, coverage, cost, and power consumption. To overcome the limitations of a single technique, hybrid methods that combine several techniques (e.g., Wi-Fi + Bluetooth) are often used [7,8,9,10,11,12], but the corresponding infrastructures may not be available in many environments.

An effective way to improve the performance of indoor positioning methods is to fuse them with spatial information such as maps, which does not require additional hardware. Indoor spaces contain constraints in the form of geometric, topological, and semantic information, which can also be used to constrain the trajectory of objects, thereby optimizing positioning results. A popular manner to utilize spatial information is map matching, which refines the preliminary positioning results with spatial information in maps or navigation models [13,14,15,16].

Map matching can be categorized into three main types: *point-to-point matching*, *trajectory matching*, and *probabilistic graphical model* [16]. The point-to-point method matches location points with the places of indoor spaces according to floor plans. Point-to-point matching is simple and computationally efficient, but it suffers from the way that the path network is digitized [17]. Trajectory matching uses the geometry and topology information of indoor structures (e.g., corners, narrow corridors, and rooms) to match the obtained trajectory [18,19]. Compared to point-to-point matching, trajectory matching is more robust and has smaller matching error, but it is more complex and has poor real-time capability. Probabilistic graphical model-based map matching calculates the location by associating each location with a probability and then updating the probability using spatial constraints [7,13,20]. Probabilistic graphical mode-based approaches can achieve higher accuracy than point-to-point matching and trajectory matching, but its computational burden is heavier.

This study focused on analyzing the effect of spatial characteristics of indoor spaces on three popular map matching methods: particle filtering (PF), hidden Markov model (HMM), and geometric methods. The first two methods are probabilistic graphical model-based methods, whereas the third one is a point-to-point matching method. These methods have been widely used for indoor positioning and navigation [7,13,21,22,23,24,25,26]. An early system using a particle filter is proposed to fuse foot-mounted inertial sensor data and spatial information in Woodman and Harle [21]. APFiLoc [22] uses an augmented PF to fuse smartphone inertial sensors, landmarks, and map information for achieving better indoor localization accuracy. In Hilsenbeck et al. [23], a PF is utilized to fuse pedometer readings with Wi-Fi measurements for mobile indoor positioning, in which indoor environments are discretized graphs to reduce computational complexity. In Zhou et al. [24], a HMM is used to realize pedestrian localization by matching the user’s trajectory that is generated by pedestrian dead reckoning (PDR) with an indoor road network. HTrack [13] also utilizes an HMM for achieving efficient indoor localization and tracking by using user’s heading information. A geometric map matching method is used to improve the positioning accuracy of PDR by detecting the collision between the pedestrian trajectory and floor map. However, most existing studies focus on refining the positioning results by improving the map matching algorithm [26].

Although several studies have demonstrated that certain map matching algorithms are affected by some spatial structures (e.g., parallel paths), they focus on the analysis of single map matching method or few spatial structures. In this study, we explored how the most commonly-used four spatial characteristics (namely forks, open spaces, corners, and narrow corridors) affect three popular map matching methods (namely PF, HMM, and geometric methods). We hope that the results will be helpful for future researchers in choosing proper map matching algorithms with considering the spatial characteristics.

The remainder of this paper is organized as follows: Section 2 presents the three map matching methods of interest, including the basic idea, advantages and disadvantages, and their applications. In Section 3, we provide a theoretical analysis on how spatial characteristics affect the performance of map matching methods. Section 4 describes our experimental setup and specific results analysis. Conclusion and future work are given in Section 5.

## 2. Map Matching Methods of Interest

**Figure 1:**
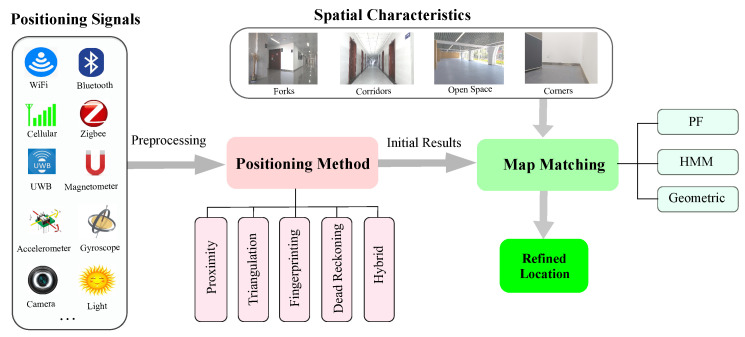
Map matching process.

In this section, we introduce the three popular map matching methods of interest, including PF, HMM, and geometric methods. We first demonstrate the relationship of map matching, spatial characteristics, and positioning techniques before elaborating each of them. As shown in Figure 1, map matching methods take as input initial positioning results and spatial constraints from indoor spaces, and output the refined location. The initial results can be obtained by using different positioning methods (e.g., proximity, triangulation, fingerprinting, and dead reckoning) according to the positioning signals (e.g., Wi-Fi, cellular, and Bluetooth).

### 2.1. Particle Filtering

Particle filtering is one of commonly-used map matching methods, which uses a set of particles to approximate the posterior distribution of some stochastic process given noisy observations. These particles are associated with a set of weights. In each round, particles propagate according to the state model, and the corresponding weights would be updated based on observations and spatial constraints. For instance, if a particle crosses a wall or lies in non-navigable areas, the weight of this particle would be set to 0. The final step is to re-sample these particles according to their weights and those whose weights are below a threshold are removed. Over time, the particles typically converge to the most likely position of the user. Map matching based on particle filtering can usually achieve relatively high positioning accuracy, but it suffers from heavy computational cost [22].

Particle filtering involves two models: state model and measurement model. In this study, we express the measurement model as $M_{k}=\left(\alpha_{k},\theta_{k}^{\prime }\right)$, where α and $\theta^{\prime }$ are the acceleration and direction angle observed by the built-in sensors of the smart phone when the pedestrian walks to the $k_{th}$ step. The state model can be expressed as $x_{k}^{i}=x_{k-1}^{i}+s_{k-1}^{i}\cdot \sin\left(\theta_{k}^{i}+v_{i}\right)$ and $y_{k}^{i}=x_{k-1}^{i}+s_{k-1}^{i}\cdot \cos\left(\theta_{k}^{i}+v_{i}\right)$, where $x_{k}$,$y_{k}$ are the coordinates of the position when the user moves to the $k_{th}$ step, $s_{k-1}$ is the pedestrian step length set in the algorithm, and θ is the walking direction obtained by the Kalman filter processing of $\theta^{\prime }$, $v_{i}$ represent Gaussian noise. In the following, we briefly introduce the process of map matching method based on particle filtering:

- **Initialization**. Draw *N* particles $\left\{x_{0}^{i},y_{0}^{i},w_{0}^{i}\right\}$$\left(i=1,2,...,N\right)$ according to the proposal probability function. $x_{0}^{i}$ and $y_{0}^{i}$ are the coordinates of the *i*-th particle, and $w_{0}^{i}$ is the initial weight of the particle which is assigned by a value of 1/N.
- **Prediction**. Calculate the state $\left(x_{k}^{i},y_{k}^{i}\right)$ at the *k*-th step for each particle according to the state model.
- **Weight update**. Update the weights of particles using the spatial or other constraints. For example, the weight of a particle will be set to zero when it crosses obstacles (e.g., walls). When the Wi-Fi fingerprinting is integrated into the PF, the particles that are closer to the estimated results from the Wi-Fi fingerprinting will be assigned larger weights. After this, the weights of particles are normalized by
  (1) $$w_{k}^{i}=w_{k}^{i}/\sum_{j\;=\;1}^{N}w_{k}^{j}.$$
- **State estimate**. The location of the user $\left\{x_{k},y_{k}\right\}$ is obtained according to the position of each surviving particle $\left\{x_{k}^{i},y_{k}^{i}\right\}$ and their weight $w_{k}^{i}$, namely
  (2) $$x_{k}=\sum_{i\;=\;1}^{N}x_{k}^{i}\cdot w_{k}^{i}$$
  (3) $$y_{k}=\sum_{i\;=\;1}^{N}y_{k}^{i}\cdot w_{k}^{i}.$$
- **Resampling**. Re-sampling is a way to avoid the degeneracy problem, i.e., that most importance weights are close to zero. More specifically, when the effective number of particle (denoted by $N_{eff}$) is below a threshold ($N_{thr}$), namely $N_{eff}=\frac{1}{\sum_{i\;=\;1}^{N}{\left(w_{k}^{i}\right)}^{2}}<N_{thr}$, then the following re-sampling operations are performed: (1) Draw *N* particles from the current particle set with probabilities proportional to their weights; (2) replace the current particle set with the new one; (3) set $w_{k}^{i}=\frac{1}{N}$ for all particles.

### 2.2. Hidden Markov Models

Hidden Markov Models (HMM) are also one of the commonly-used map matching methods for indoor positioning. They can usually provide excellent accuracy, but suffer from high computational cost. A HMM model is determined by a transition probability matrix *A*, an emission probability matrix *B*, and an initial probability distribution π. Thus, given an observation sequence $O=\{o_{1},\cdots ,o_{M}\}$, the model λ=(A,B,π), and the hidden states $S=\{s_{1},\cdots ,s_{N}\}$, we can use the Viterbi algorithm to infer the location result with the highest probability. Before the location is inferred, we need to determine the transition probability matrix and emission probability matrix [27].

**Figure 2:**
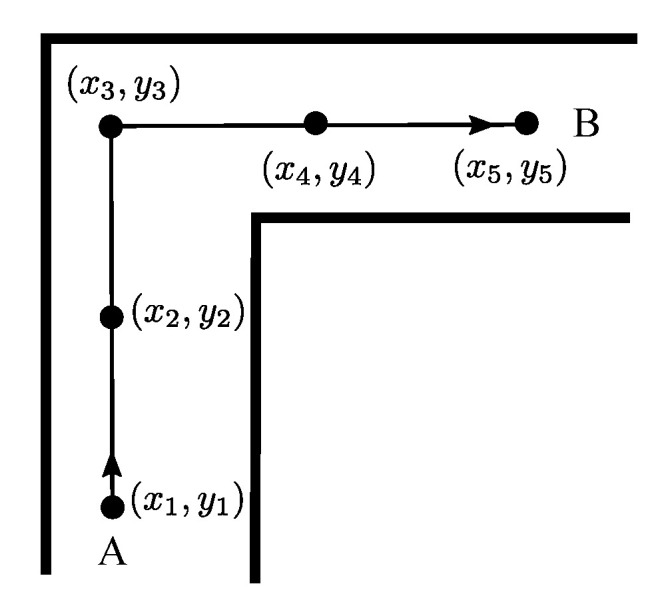
Example of illustrating three distance metrics (Euclidean distance, path distance, and constant distance).

**Transition probability**. The transition probability matrix *A* can be formed as follow:

(4) $$a_{ij}=\frac{\frac{1}{D_{ij}}}{\sum_{k}^{}\frac{1}{D_{ik}}}$$

where $a_{ij}$ is the probability of transiting from location *i* to location *j*. $D_{ij}$ is the distance from *i* to *j*, *k* is the number of points that can be reached from *i*. In this study, we considered three distance metrics, namely Euclidean distance $d_{ED}$, shortest path distance $d_{PD}$, and constant distance $d_{CD}$. Euclidean distance is the straight line distance between two points. The shortest path distance refers to the shortest distance along the walkable path in the indoor space. The constant distance refers to that in the positioning process, each position update can move a constant distance at most. To better describe the difference between the three metrics, we provide a graphical example to illustrate it. As shown in Figure 2, suppose that a user walks from point A $\left(x_{1},y_{1}\right)$ to point B $\left(x_{5},y_{5}\right)$, and during which he needs to pass points $\left(x_{2},y_{2}\right)$, $\left(x_{3},y_{3}\right)$, and $\left(x_{4},y_{4}\right)$, respectively. Thus, the three distance metrics can be expressed as:

(5) $$d_{ED}=\sqrt{{\left(x_{5}-x_{1}\right)}^{2}+{\left(y_{5}-y_{1}\right)}^{2}}$$

(6) $$d_{PD}=\sum_{i=2}^{5}\sqrt{{\left(x_{i}-x_{i-1}\right)}^{2}+{\left(y_{i}-y_{i-1}\right)}^{2}}$$

(7) $$d_{CD}=n\cdot \beta$$

where *n* denotes the number of steps (here is 4), β is a constant distance for each step. Accordingly, the HMM using the three distance metrics is denoted by HMM-ED, HMM-PD, and HMM-CD, respectively. Note that ED, PD, and CD denote Euclidean distance, path distance, and constant distance.

**Emission probability**. The emission probability matrix *B* can be formed as follows:

(8) $$b_{j}\left(k\right)=P\left(o_{k}|s_{j}\right),k=1,2,\cdots ,M;j=1,2,\cdots ,N$$

where $b_{j}\left(k\right)$ is the probability of generating the observation $o_{k}$ at location $s_{j}$. After calculating the transition matrix and emission matrix, we can then use the Viterbi algorithm to infer the location of the user. More details about the location inference can be found in Gu et al. [27].

### 2.3. Geometric Method

The geometric algorithm is a traditional map matching method, the basic idea of which is to map positioning results with the path network. It first determines the currently occupied path segment by certain rules, and then calculates the current location on the selected path segment [28,29]. These rules involve three weights, namely distance, orientation, and adjacent weight.

The distance weight is based on the distance between the pedestrian and the navigation path, which is described as:

(9) $$w_{d}=\frac{a}{d}$$

where *a* is the weight adjustment coefficient and *d* is the Euclidean distance from the initial positioning point to its projection point on each navigation path.

The orientation weight is obtained by the consistency between pedestrian travel direction and navigation path direction, namely

(10) $$w_{o}=\frac{b}{\cos\alpha }$$

where *b* is a weight adjustment coefficient, and α is the angle between the pedestrian direction reported by the smartphone compass and the direction given by the navigation path.

The adjacent weight is calculated according to the distance of the adjacent trajectory points, namely

(11) $$w_{a}=\left\{\begin{array}{c} 1,\;d_{a}\leq c\\ 0,\;d_{a}>c \end{array}\right.$$

where $d_{a}$ is the distance between the projection point of the initial positioning point on the navigation path and the previous positioning point. Note that *a*, *b*, and *c* are determined empirically, and are set to 3, 3, and 2 in this study, respectively.

Based on the above three weights, we can get a total weight, namely,

(12) $$W=w_{d}+w_{o}+w_{a}.$$

Thus, the path with the largest total weight is chosen as the occupied path, and the projection of the initial positioning point on the path is the final positioning result. Although the geometric algorithm is simple and computationally efficient, it often suffers from poor accuracy.

## 3. Theoretical Analysis of Spatial Characteristics for Map Matching

The performance of map matching algorithms depends highly on the spatial characteristics of indoors spaces. The way that different map matching methods deal with initial positioning results also differ. In this section, we provide a theoretical analysis on how different types of spatial characteristics affect the performance of the chosen three map matching methods (namely PF, HMM, and geometric methods). We present the results of our experimental verification of the analysis in next section.

### 3.1. Fork

**Figure 3:**
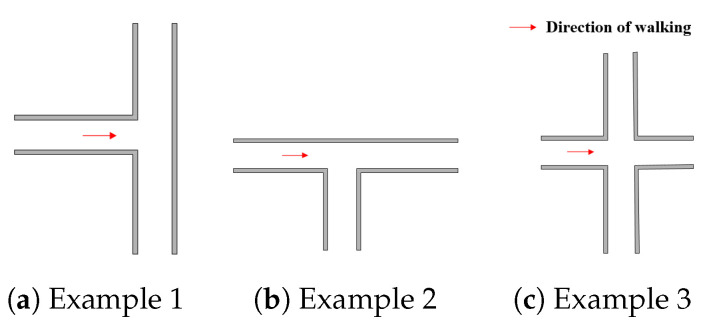
Examples of forks.

**Figure 4:**
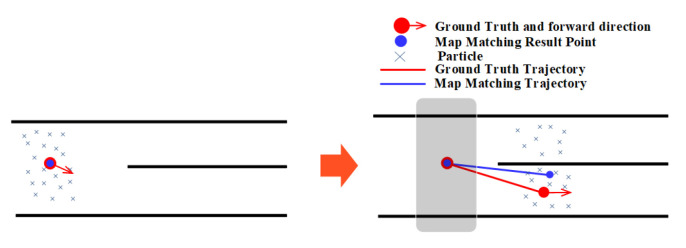
The effect of fork on the particle filtering (PF) (example 1).

**Figure 5:**
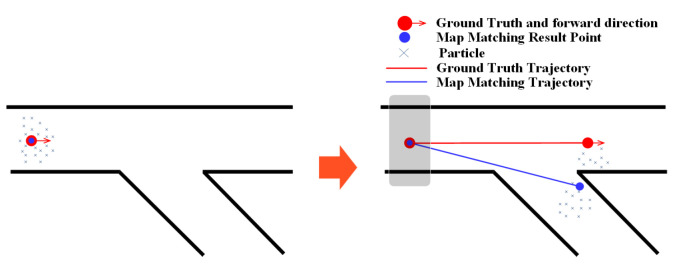
The effect of fork on the PF (example 2).

A fork in indoor environments is the point or place at which the walking path branches into two or more due to walls or other obstacles. Figure 3 shows some examples of forks. Forks have an impact on the performance of map matching methods. The PF estimates the location of the user by averaging particles with corresponding weights. We analyzed the effect of the fork on the PF using two examples. The first example is shown in Figure 4, where a few particles move to the wrong path at the fork, but the majority of particles forward along the correct path and hence the estimated result is on the right path with a certain error that is caused by the particles moved to the wrong path. The second example is that the majority of particles forward to the wrong path, as demonstrated in Figure 5, resulting in that the estimated result is on the wrong path. This can further make the algorithm get stuck at the fork, and cause failure for subsequent estimates.

**Figure 6:**
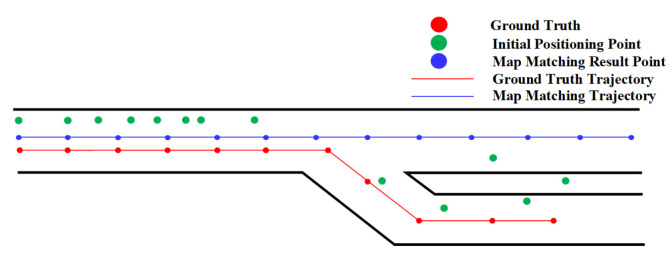
The effect of fork on the Hidden Markov Model (HMM).

The HMM algorithm matches the positioning results according the transition probability and emission probability, which are affected by forks. Suppose that the user has traveled a certain distance before reaching a fork. It is possible that the matching trajectory cannot reach the fork because of the accumulated error. As demonstrated in Figure 6, the HMM may match the positioning results near the fork to the wrong straightforward path rather than the actual path on the right side. This is because the matched results are far away from the ground truth location before the fork due to the accumulated error. After the fork, its transition probability to the wrong path is greater than its transition probability to the correct path. Also, the two paths after the fork have the similar direction and hence share similar emission probability. Therefore, according to the principle of the maximum probability of HMM algorithm, it will lead to the wrong path and result in a large positioning error.

**Figure 7:**
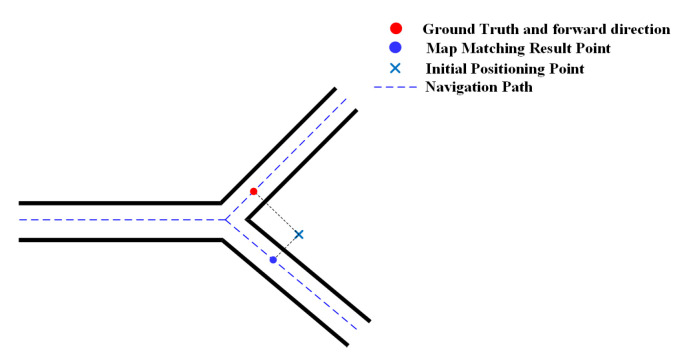
The effect of fork on the geometric algorithm.

The effect of fork on the geometric algorithm is illustrated in Figure 7. The geometric method may match the positioning results to a wrong path when there exists a certain error in the initial positioning results near the fork, and the walking directions before and after the fork are the same.

### 3.2. Open Space

Open spaces refer to large indoor areas (e.g., stadium, gym, or station) where there are no/few spatial constraints that can be used to constrain users’ movement. The PF method makes use of spatial constraints to improve the positioning accuracy by discarding these particles that violate spatial constraints. Since there are no spatial constraints available in open areas, the PF algorithm will fail to improve the positioning results. By contrast, the HMM algorithm can continue to improve the initial positioning results to some extent in open spaces by allowing only transitions between adjacent points in the trajectory, which may somehow avoid the jumping problem of initial positioning results. For the geometric algorithm, the lack of space constraints makes the algorithm perform poorly in open areas. This is because there are too many accessible paths along all directions in open spaces, making the geometric method unable to select the correct candidate path segment through the direction weight. In addition, when the initial positioning error is larger than the path segment interval, the proximity weight cannot calibrate the positioning results to the correct path. Although the connection weight can improve the positioning accuracy, in general, open spaces have a negative impact on the geometric algorithm.

### 3.3. Corner

**Figure 8:**
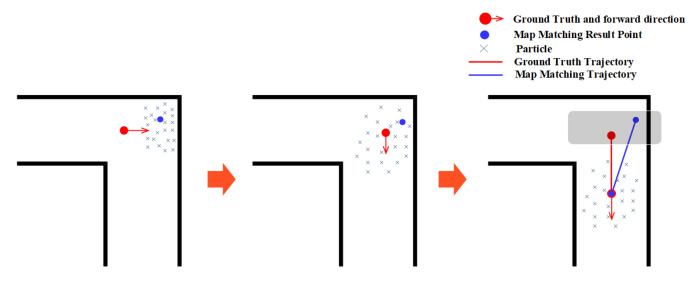
The effect of corners on the PF.

A corner refers to a place where the path has rotated a certain angle in the direction of pedestrians. In the PF algorithm, corners can quickly help eliminate the invalid particles that cross walls, and hence result in a better accuracy by keeping track of correct particles, as shown in Figure 8. In general, the more corners that exist in the environment, the better the accuracy of PF.

**Figure 9:**
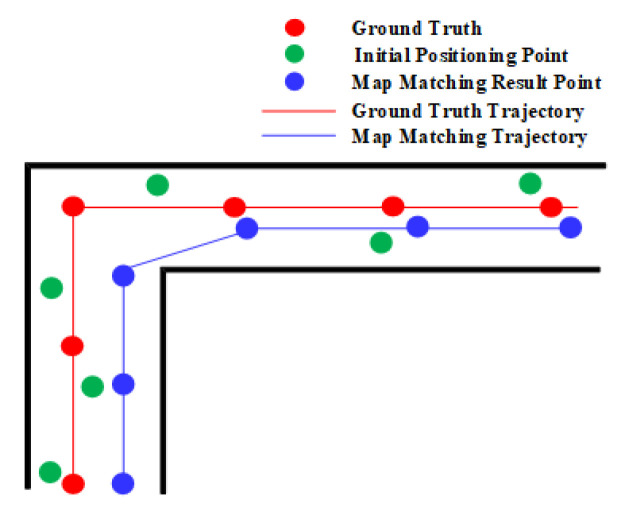
The effect of corners on the HMM.

The HMM often benefits from the spatial constraints imposed by corners. Figure 9 shows an example of the HMM improving the positioning results, from which we can see that the increasingly accumulated error before the corner can be significantly reduced by using heading constraints imposed by the corner. Corners provide both heading constraints and distance constraints for the HMM, which are usually helpful in improving the positioning accuracy.

**Figure 10:**
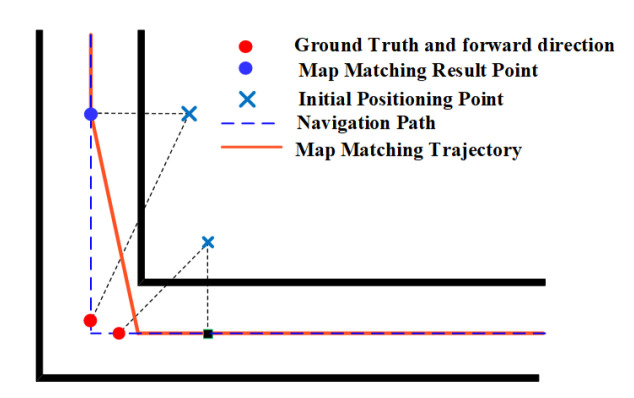
The effect of corner on the geometric method.

Corners also have a positive effect on the geometric algorithm. As shown in Figure 10, the matched positioning error is large before a corner, but the geometric algorithm significantly reduces the error by matching the positioning result to the projected point on the right edge. The heading constraints imposed by corners enable the geometric algorithm to use such information to correct positioning results.

### 3.4. Narrow Corridor

**Figure 11:**
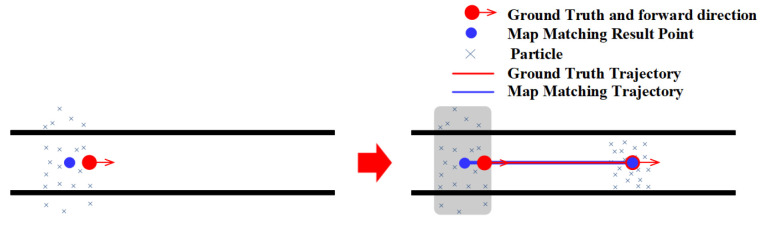
The effect of narrow corridor on the PF.

Narrow corridor refers to a narrow aisle surrounded by walls, which can provide heading constraints and boundary constraints. Narrow corridors help the PF improve the location estimation accuracy by limiting particles to forward along the walking direction and eliminating these particles that cross walls. As shown in Figure 11, the particles are usually restricted to move along the walking direction in a narrow corridor, and these particles that move to the wrong direction will soon hit/cross the walls on both sides of the corridor, which will be eliminated in the state estimation stage. Only particles with the movement direction similar to the user’s walking direction are left and used to calculate the user’s location. This will make the PF result in a better accuracy.

**Figure 12:**
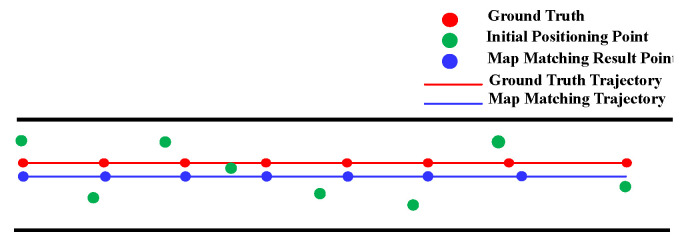
The effect of narrow corridor on the HMM.

Narrow corridor is also helpful in improving the performance of HMM. It is observed that people usually walk along the middle line of the corridor and initial positioning results are generally distributed on the two sides of the ground truth trajectory due to measurement noise, as shown in Figure 12. A common practice of setting the location states of HMM in narrow corridors is to set them along the middle line of the corridors. By matching the initial positioning results to the location states of HMM, we are able to make better location estimates that are closer to the ground truth locations.

**Figure 13:**
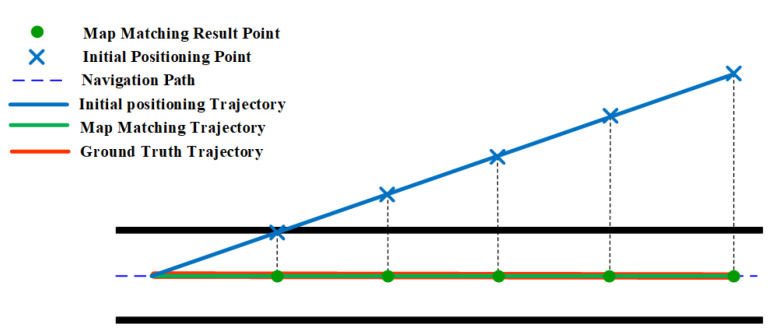
The effect of narrow corridor on the geometric algorithm.

Similar to the HMM, the geometric algorithm also benefits from narrow corridors which can somehow limit the error of location estimates. As shown in Figure 13, the geometric algorithm is able to match the initial positioning results to the right path event the accumulated error is increasing. This is because there is often a single navigation path in the relatively narrow narrow corridor and users walk generally along the middle line of the corridor.

## 4. Experiments and Results

### 4.1. Experiment Setup

**Figure 14:**
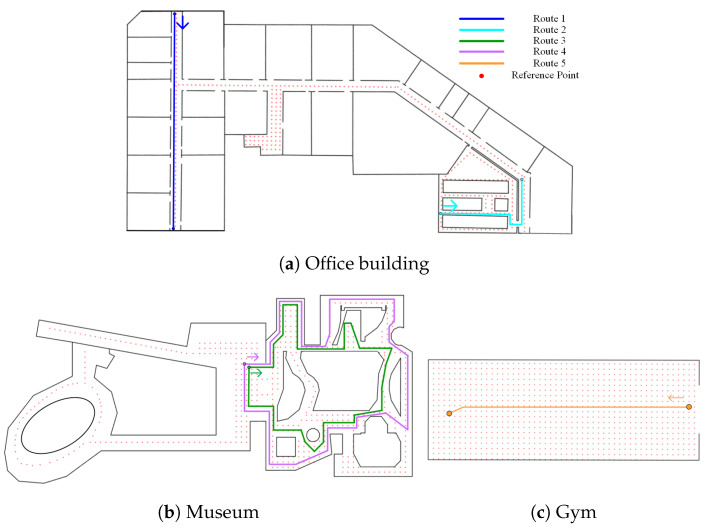
Floor plan of experimental scenarios.

We evaluated the effect of different spatial characteristics on the three map matching methods, namely PF, HMM, and geometric method. Three common indoor scenarios were considered, an office building, a museum, and a gym. Figure 14 shows the floor plans of the selected experimental scenarios. The layout of the office building was similar to that of hotels and apartments, containing corridors with multiple turns, and rooms of different sizes on both sides of the corridor. The museum was similar to many shopping malls, including some open spaces, and corridors with multiple shapes. The gym was like many stations or stadiums that feature large open spaces.

Five routes, which consist of different spatial characteristics, were designed to analyze the effect of these spatial characteristics on the selected map matching methods. The length of the five routes was 51, 33, 114, 138, and 27 m, respectively. As shown in Figure 14, we designed two routes (route 1 and 2) in the office building. Route 1 consists of a narrow corridor and was mainly used to analyze the effect narrow corridor on the map matching methods. Route 2 was used to analyze the effect of fork on the map matching methods. There were two routes (route 3 and 4) designed in the Museum. Routes 3 and 4 were mainly used to analyze the effect of corners and turns. Route 5 in the gym was utilized to analyze the influence of open spaces on the map matching algorithms.

To compute the initial positioning results, two users were asked to walk at a constant speed along the designed routes, and the data from Wi-Fi, accelerometer, magnetometer, and gyroscope were sourced from built-in detectors in a Google Nexus smartphone. We have set a number of markers along the routes. In order to evaluate the positioning accuracy, users need to press a button on the smartphone when they walk through these markers so that the corresponding timestamps are recorded. Thus, we can get the ground truth locations at each walking step by interpolating based on these markers’ locations and corresponding timestamps of encountering them. During the experiments, radio maps (or fingerprint database) were collected along the designed routes with an interval of 1 m, which were used to provide initial positioning results for the HMM and weight updating constraints for the PF. Each fingerprint consists of a vector of received signal strength (RSS) from all visible access points (APs) and the corresponding location.

### 4.2. Algorithm Implementation

We implemented the selected map matching algorithms in Matlab. For the PF algorithm, we considered two different settings with different number of particles per setting, namely 500 and 1000 particles, respectively. The PF algorithm uses acceleration readings and heading to forward particles and update the weights by the positioning results estimated from Wi-Fi fingerprinting and/or spatial constraints. For the HMM algorithm, we used the Wi-Fi fingerprinting to calculate the initial positioning results, which were subsequently matched according to the transition matrix and emission matrix of the HMM. We considered three distance metrics for calculating the transition matrix, namely Euclidean distance, path distance, and constant distance. For the geometric algorithm, the PDR method was used to compute the initial positioning results.

### 4.3. Overall Positioning Results

We first show the positioning results of the three map matching methods on five routes. As can be seen from Table 1, spatial constraints can significantly improve the positioning accuracy of the PF on five routes. This is best witnessed on route 4 where the PF without spatial constraints achieve an error of about 8 m, which is reduced to about 3.3 m with spatial constraints.

Table 2 shows the initial positioning results from the Wi-Fi fingerprinting and the improved positioning results of HMM with different distance metrics. It is also can be observed that the HMM with path distance can generally perform better than that with constant distance or Euclidean distance though the HMM with constant distance has a smaller positioning error on route 4.

Table 3 shows the positioning error of PDR method and that improved with the geometric algorithm. It can be found that the the geometric map matching method can significantly improve the positioning error over the PDR for most routes except for route 5, which is an open space area and there are no any spatial constraints.

In the following sections, we elaborate on how each type of spatial characteristic affects the three map matching methods.

### 4.4. Effect of Fork

**Figure 15:**
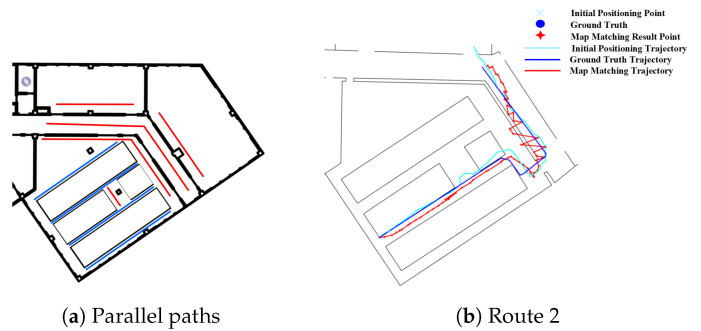
Positioning results of the PF algorithm on Route 2 (Forks).

In this section, we analyze the effect of fork on the three map matching methods. As we have described in Section 3.1, forks will cause multiple parallel paths. Figure 15a demonstrates that there are multiple parallel paths in the experimental environments, in which the red line segments are a set of parallel paths, the blue line segments are another set of parallel paths.

**Figure 16:**
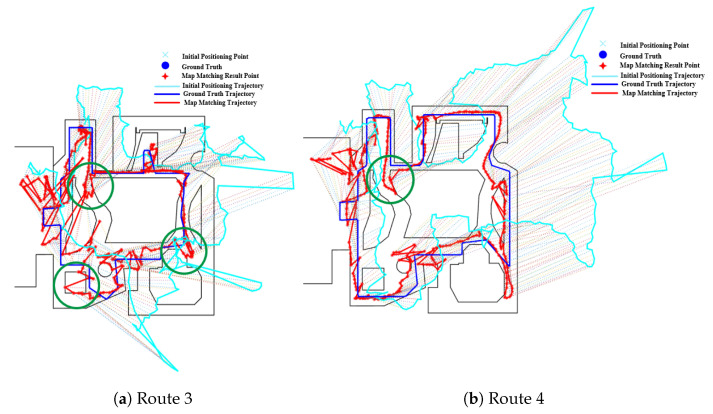
Positioning results of the PF algorithm on Routes 3 and 4 (Forks).

Figure 15b shows the effect of fork on the PF algorithm, from which we can see that the path on the right of the route 2 is matched to a incorrect parallel path and there are multiple jumps in the estimated position between the parallel paths, thus leading to a relatively large error. This is mainly due to the existence of multiple paths caused by forks. The effect of forks in Routes 3 and 4 on the PF method is also illustrated in Figure 16, where the green circle denotes the region of forks. It can seen that the forks often lead to a larger positioning error.

**Figure 17:**
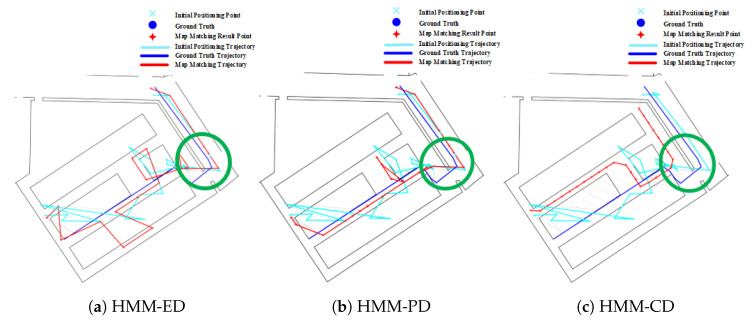
Positioning results of HMM algorithms on Route 2 (Green circle denotes the effect of forks).

The effect of forks in route 2 on the HMM is shown in Figure 17. As we have described before, the HMM algorithm with lower transition probability is prone to generate cumulative error, which makes it re-match or mismatch to the adjacent and similar paths when encountered forks. We can see that the matched results from HMM-ED and HMM-PD in the fork area (denoted by the green circle) are on the right path (see Figure 17a,b), but the matched results from the HMM-CD (see Figure 17c) are on the wrong path due to the accumulated error.

**Figure 18:**
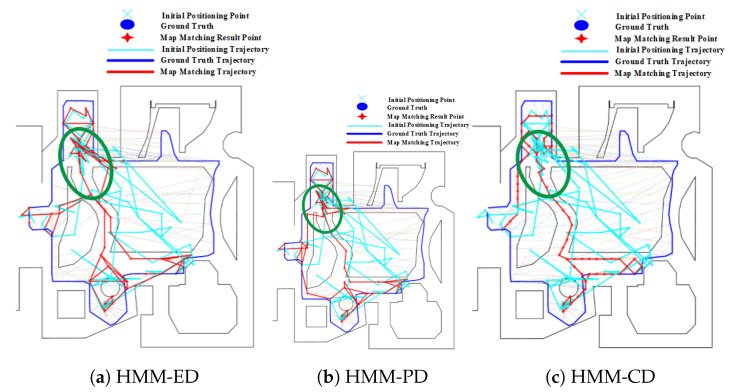
Positioning results of HMM algorithms on Route 3 (Green circle denotes the effect of forks).

**Figure 19:**
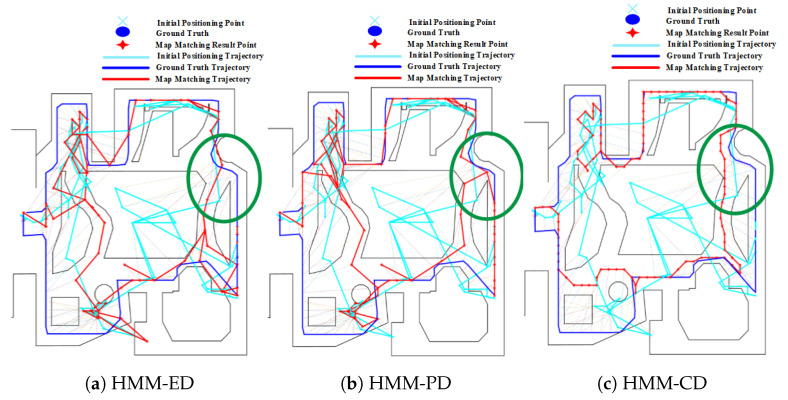
Positioning results of HMM algorithms on Route 4 (Green circle denotes the effect of forks).

The mismatching of HMM, which is caused by forks, also occurs on routes 3 and 4, as shown in Figure 18 and Figure 19. For route 3, we can see that the trajectory was mistakenly matched to the middle path, leading to a large positioning error in the area. However, this error can be corrected by increasing the length and range of trajectories, as shown in Figure 19 on route 4. Due to the range of trajectories is increased, mismatching would lead to very low emission probability. Therefore, the HMM will re-match the entire trajectory and correct it to the right path. Therefore, the overall positioning accuracy on route 4 is higher than that on route 3.

**Figure 20:**
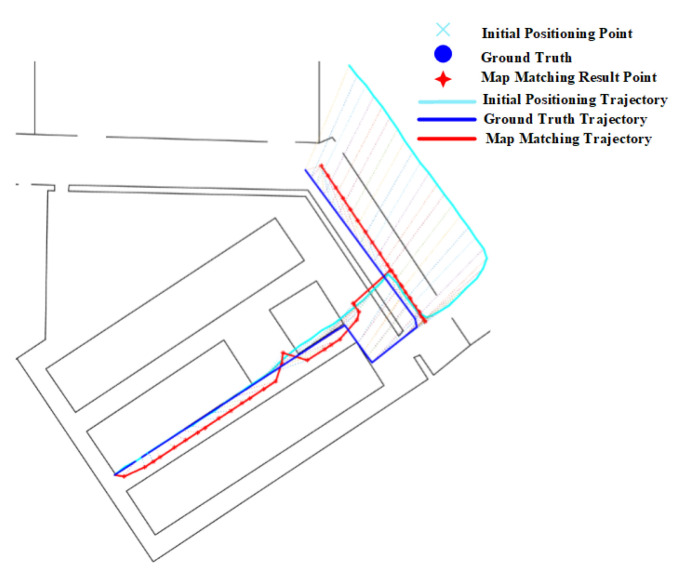
Positioning results of the geometric algorithm on Route 2 (Forks).

**Figure 21:**
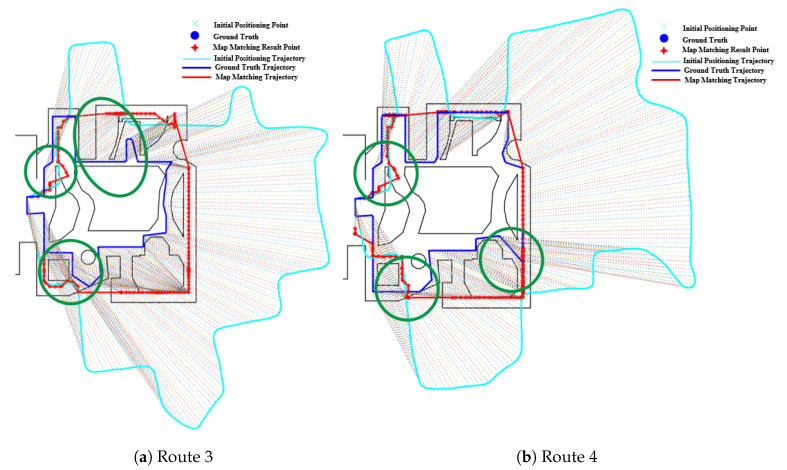
Positioning results of the geometric algorithm on Routes 3 and 4 (Green circle denotes the effect of forks).

For the geometric algorithm, it is obvious from the experimental results that the performance of the geometric algorithm on route 2 is also poor. As shown in Figure 20, due to the impact of parallel paths caused by the fork (Figure 15b), part of Route 2 is positioned on the adjacent parallel path. From the results on routes 3 and 4 in Figure 21, We can also clearly see that forks have a greater negative impact on the geometric algorithm, often leading to a large positioning error.

### 4.5. Effect of Open Space

**Figure 22:**
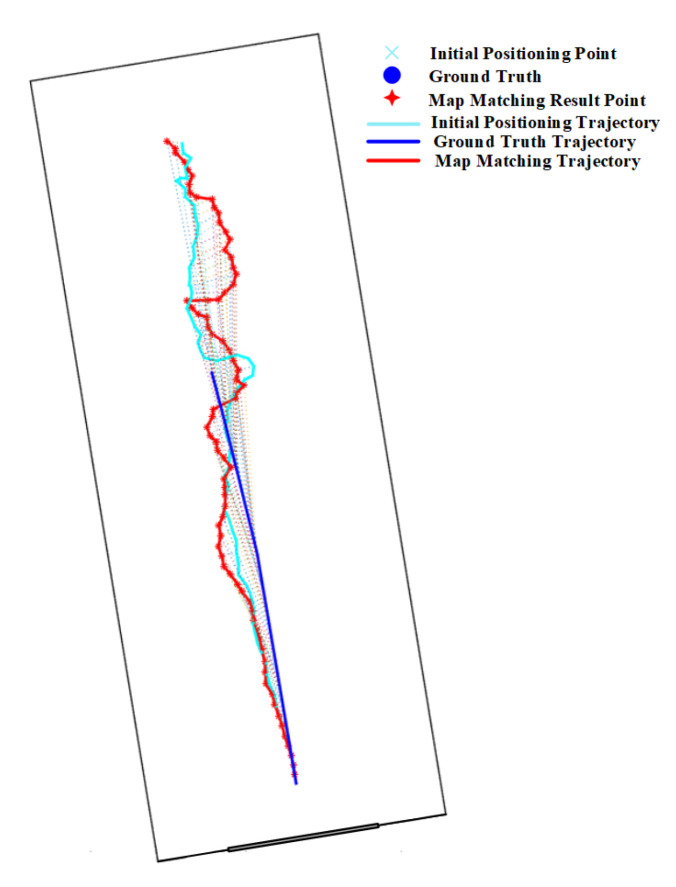
Positioning results of the PF algorithm on Route 5 (Open Space).

**Figure 23:**
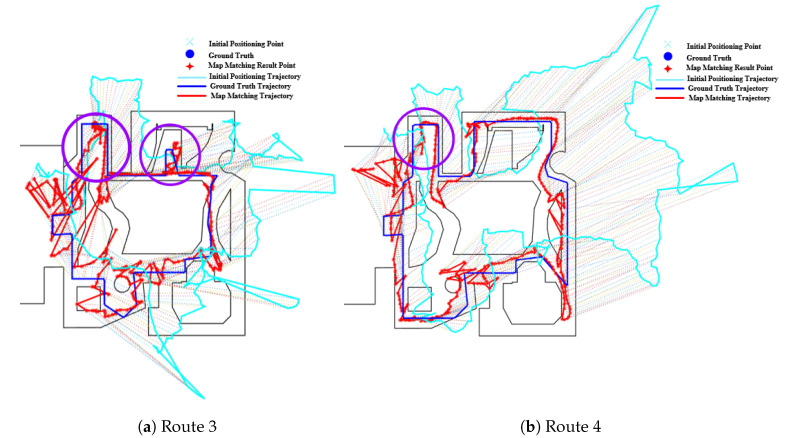
Positioning results of the PF algorithm on Routes 3 and 4 (Purple circle denotes the effect of Open Space).

The effect of open space on the PF is shown in Figure 22, from which we can see that the PF performs very poorly on route 5 due to the lack of spatial constraints, and the improvement of the initial positioning results is very limited. It can be observed from Figure 22 that the matched trajectory deviates significantly from the ground truth trajectory as the accumulated error increases. The negative effect of open spaces on the PF can be also found on routes 3 and 4 in Figure 23, where the open space areas (denoted by purple circles) often witness a larger positioning error and the matched trajectory deviate obviously from the ground truth one.

**Figure 24:**
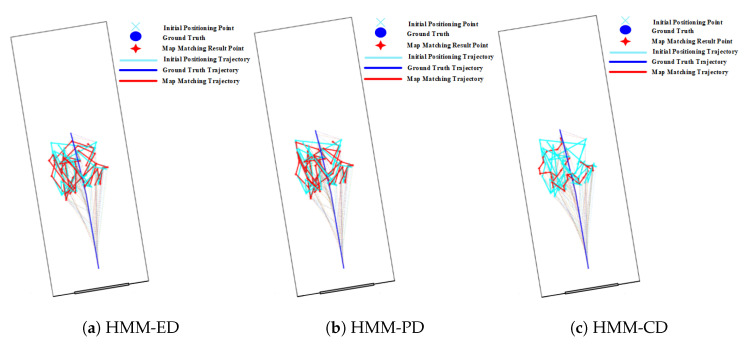
Positioning results of the HMM algorithm on Route 5 (Open Space).

Figure 24 demonstrates the effect of open space on the HMM algorithm, from which we can see that open spaces have marginal impact on the HMM. The matched trajectory is almost the same of the initial trajectory due to the lack of spatial constraints.

**Figure 25:**
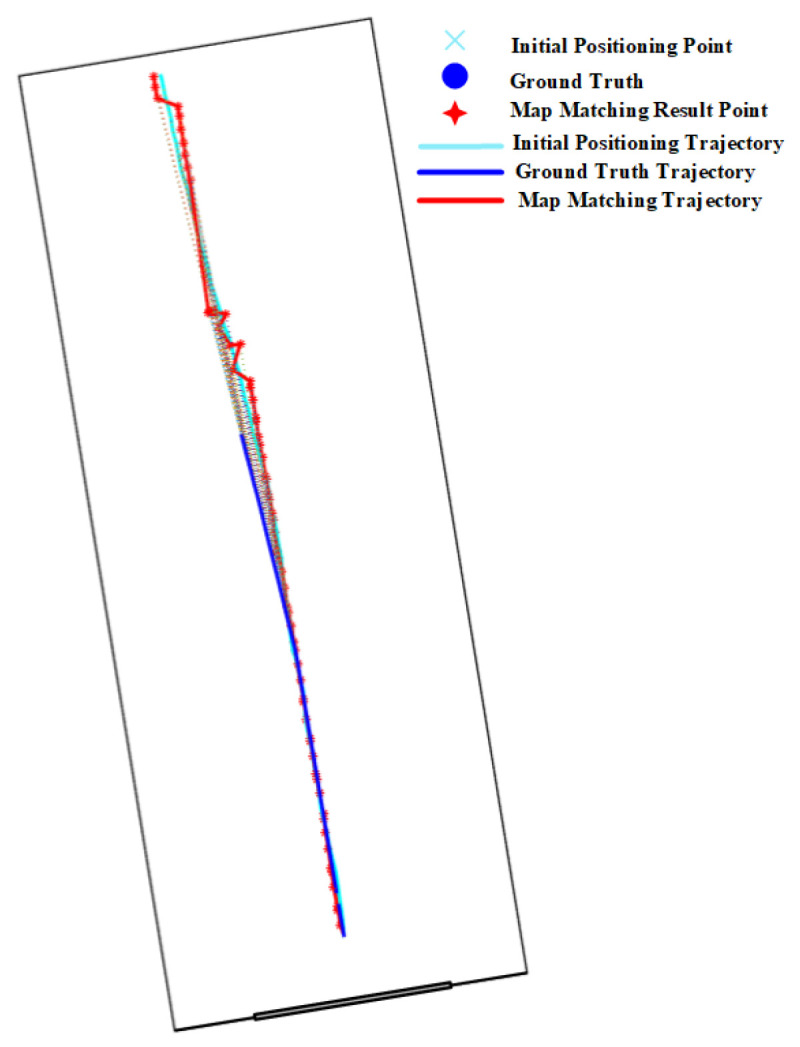
Positioning results of the geometric algorithm on Route 5 (Open Space).

**Figure 26:**
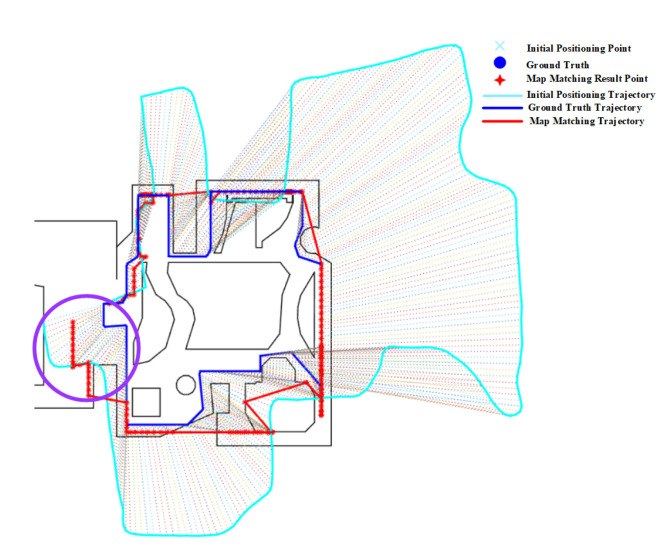
Positioning results of the geometric algorithm on Route 4 (Purple circle denotes the effect of Open Space).

Open spaces have also no much impact on the geometric algorithm, as shown in Figure 25. The matched results of the geometric algorithm are usually on a navigation path close to the initial positioning results due to the accumulated error, and are easy to drift to incorrect areas. It is also obvious to see from Figure 26 that open space areas (denoted by purple circles) encounter a larger positioning error than narrow areas.

### 4.6. Effect of Corner

**Figure 27:**
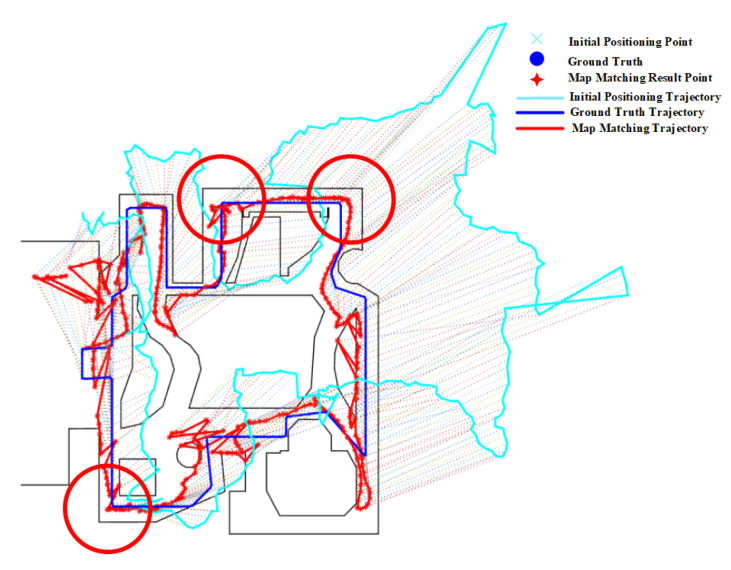
Positioning results of the PF algorithm on Route 4 (Red circle denotes the effect of Corners).

The effect of corners on the PF algorithm is demonstrated in Figure 27. It can be seen that the positioning error of PF is significantly reduced after passing corners. This is because corners can provide useful spatial constraints by removing invalid particles that cross walls or other obstacles.

**Figure 28:**
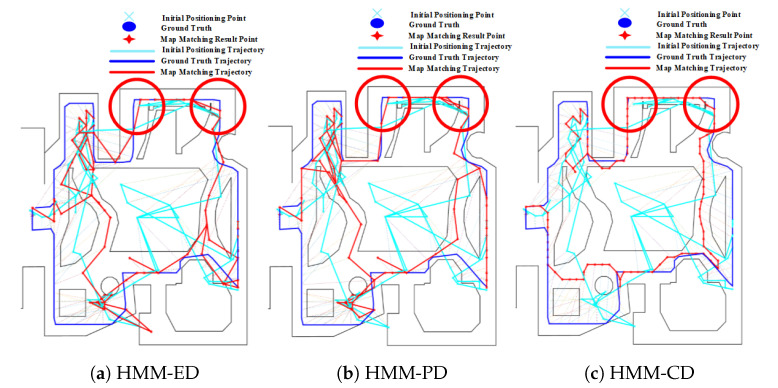
Positioning results of the HMM algorithm on Route 4 (Red circle denotes the effect of Corners).

Similar to the PF, the HMM also benefits from corners. As shown in Figure 28, corners (denoted by red circles) help reduce the positioning error by matching initial results to the correct path.

**Figure 29:**
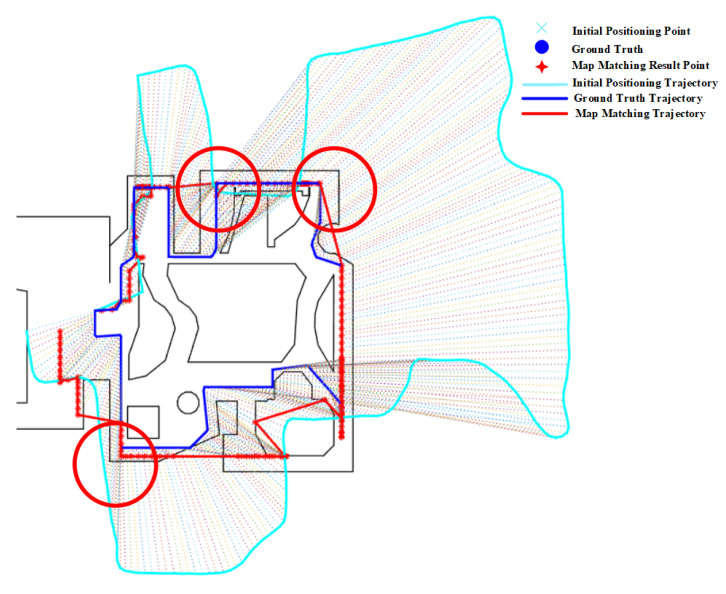
Positioning results of geometric algorithm on Route 4 (Red circle denotes the effect of Corners).

Corners are also beneficial for the geometric algorithm. As demonstrated in Figure 29, the initial results from PDR suffer from large positioning errors. By using spatial constraints imposed by corners, the geometric algorithm can significantly improve the positioning accuracy.

### 4.7. Effect of Narrow Corridor

As shown in Table 1, the accuracy of map matching methods on route 1 is better than that on other routes. This is because route 1 contains a long narrow corridor, which is very helpful in reducing the positioning error.

**Figure 30:**
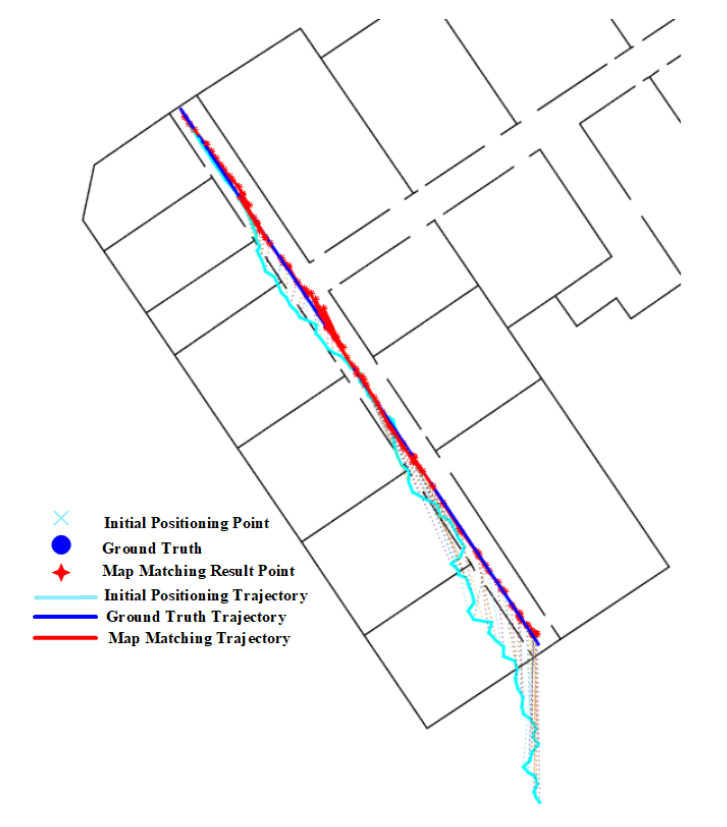
Positioning results of the PF algorithm on Route 1 (Narrow corridor).

Figure 30 shows that the initial positioning results are significantly improved by the PF with spatial constraints imposed by walls on the two sides of narrow corridors.

**Figure 31:**
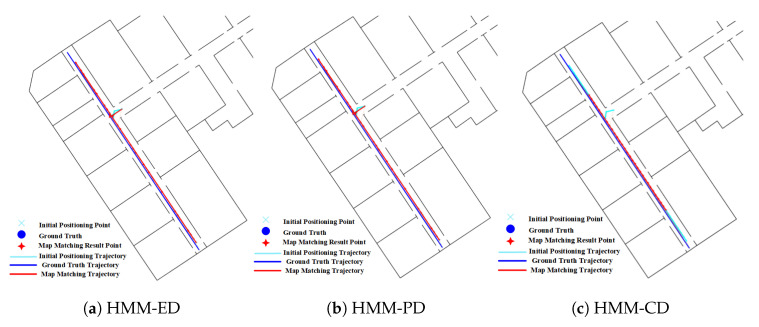
Positioning results of the HMM algorithm on Route 1 (Narrow corridor).

The positioning results on routes 1 in Table 2 show that narrow corridors have a positive effect on the HMM with path distance and Euclidean distance, but do not benefit the HMM with constant distance. The trajectories of the HMM with different distance metrics are shown in Figure 31. We can see that the matched trajectory made by the HMM with constant distance (HMM-CD) is much shorter than the initial trajectory and ground truth trajectory due to the error between the constant distance and the real transition distance. By contrast, the trajectories from the HMM-PD and HMM-ED are closer to the ground truth trajectory.

**Figure 32:**
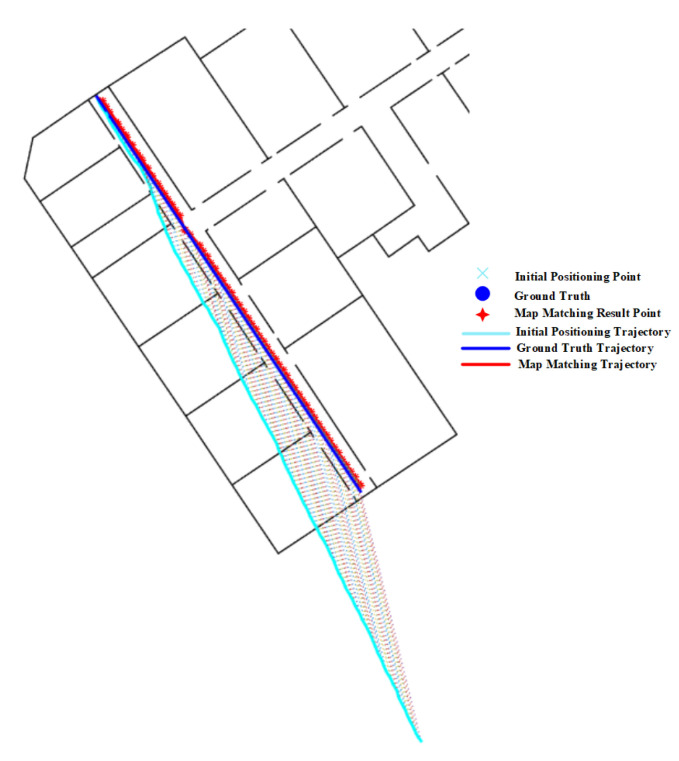
Positioning results of the geometric algorithm on Route 1 (Narrow corridor).

As shown in the experimental results on route 1 in Table 3, we can see that the geometric algorithm has greatly improved the initial positioning accuracy given by the PDR. The initial trajectory and matched trajectory are shown in Figure 32, it can be seen that the matched trajectory is very close to the ground truth trajectory, though the initial trajectory has a great deviation from the ground truth one.

### 4.8. Effects of Walls on Map Matching Algorithms

Apart from the above four types of spatial characteristics, we also analyzed the effect of walls. Indoor spaces are separated by walls and walls are often considered as the most important elements of an indoor map. Map matching algorithms use the constraints from walls to refine the coarse positioning results. Since the mechanism of each map matching algorithm is different, walls have different effects on varying map matching algorithms.

In PF algorithm, the role of walls is to eliminate invalid particles that cross walls. The weights of these invalid particles are set to 0. As shown in Figure 15 and Figure 16, when particles pass through a fork in the road, the wall separates the particles into multiple spaces. In the process of particle movement, as the particle hits the wall, it will continue to die out and supplement the weights of particles in different spaces that change constantly. In this case, the positioning results will be biased to the space the particles have greater weight, it leads to positioning error and the phenomenon of trajectory passing through the wall. As shown in Figure 27 and Figure 30, in corners and narrow corridors, the positioning accuracy is improved since walls can eliminate many wrong particles.

For the HMM algorithm, the topological relationship imposed by walls can avoid the phenomenon of passing through the wall by affecting the transfer probability of HMM, so as to improve the positioning accuracy. As shown in Figure 17, Figure 18 and Figure 19, the wall separation leads to multiple possible forward paths at the fork. The distance from the previous positioning point to multiple wrong positioning points will be less than that to the correct positioning points. At this time, the transfer probability to the wrong positioning point is greater than that to the correct positioning point, so it is easy to generate positioning errors. As shown in Figure 28 and Figure 31, walls limit the probability of transfer to the wrong location at corners and narrow corridors, which leads to a reduction in positioning errors.

In the geometric algorithm, different navigation paths are generated according to the shape of the interior space separated by walls, and the walls realize the constraint on the coarse positioning results through the navigation path. As shown in Figure 20 and Figure 21, the layout of walls is complex, and there are multiple navigation paths. In such case, the positioning results are easy to be matched to the wrong path, which lead to large positioning errors and the phenomenon of trajectory passing through the walls. As shown in Figure 32, at the narrow corridor, there is usually only a single navigation path, the positioning error will be small. As shown in Figure 29, at the corner, due to the constraint of walls, the cumulative positioning error is corrected, and the positioning accuracy is improved.

## 5. Conclusions

In this paper, we first provide a theoretical analysis on the effect of four types spatial characteristics (namely forks, open spaces, corners, and narrow corridors) on the three popular map matching methods (PF, HMM, and geometric). Then, we conducted a series of experiments in three different environments to evaluate these effects. We found that corners and narrow corridors are usually helpful in improving the positioning accuracy, while forks and open spaces often lead to a larger positioning error. It is also observed that the PF achieves better accuracy than the HMM and geometric methods. For the HMM, the path distance is more helpful than the Euclidean distance and constant distance as it better represents real movement transitions. In the future, we will extend our work by considering more types of spatial constraints and more map matching algorithms (e.g., Bayesian grid update).

## Figures and Tables

**Table 1:** Positioning errors (m) of PF with and without spatial constraints (SC).

Route No.	PF (500)		PF (1000)	
	Without SC	With SC	Without SC	With SC
1	3.53	1.48	3.31	1.48
2	1.40	1.17	1.44	1.17
3	7.72	3.43	7.91	3.36
4	7.87	3.29	8.04	3.27
5	7.79	7.81	7.83	7.72

**Table 2:** Positioning errors (m) of Wi-Fi fingerprinting and the HMM with different distance metrics where ED, PD, and CD represent Euclidean distance, path distance, and constant distance, respectively.

Route No.	Wi-Fi Fingerprinting	+HMM (ED)	+HMM (PD)	+HMM (CD)
1	3.33	3.18	3.18	5.31
2	2.69	2.69	2.42	3.88
3	5.76	6.04	6.04	6.55
4	4.01	3.64	3.50	2.81
5	8.60	8.56	8.55	8.34

**Table 3:** Positioning error (m) of PDR and the geometric algorithm. (—denotes not applicable).

Route No.	PDR	PDR + Geometric
1	9.08	5.89
2	3.43	1.98
3	19.05	9.91
4	18.53	7.18
5	10.89	−
